# Improvement of Phenolic Compound and Anthocyanin Extraction Efficiency From *Clitoria ternatea* by Autogenous Pressurization Method in a Sealed‐Vessel and Determination of Anthocyanin Compounds by LC‐HRMS

**DOI:** 10.1002/fsn3.71649

**Published:** 2026-03-15

**Authors:** Kha Duyen Nguyen, Thi Thu Tra Tran, Thi Anh Dao Dong

**Affiliations:** ^1^ Department Food Technology, Faculty of Chemical Engineering Ho Chi Minh City University of Technology (HCMUT), VNU HCMC Ho Chi Minh City Vietnam

**Keywords:** antioxidant capacity (DPPH), autogenous pressurization method (CVE‐AP), closed‐vessel extraction, *Clitoria ternatea*, LC‐HRMS, total anthocyanin content, total phenolic content

## Abstract

*Clitoria ternatea* L. (butterfly pea) is an edible tropical flower widely used as a natural colorant and a source of anthocyanins and phenolic compounds in food applications. This study aimed to optimize the extraction of anthocyanins (TAC) and improve total phenolic content (TPC), antioxidant capacity (DPPH) from *Clitoria ternatea* flowers using the autogenous pressurization method in a sealed vessel, also known as closed‐vessel extraction under autogenous pressure (CVE‐AP). Single‐factor experiments were first performed to investigate the effects of temperature, ethanol concentration, extraction time, and liquid‐to‐solid ratio. Based on these results, the three most influential factors: autogenous pressure, time, and liquid‐to‐solid ratio were modeled using response surface methodology (RSM) with a central composite design (CCD). The optimal extraction conditions were identified as 28.2 psi, 36 min and a liquid‐to‐solid ratio of 18:1. Under these conditions, the experimental values achieved were TAC 2.72 ± 0.18 mg/g dry matter, TPC 56.82 ± 2.16 mg GAE/g dry matter, and antioxidant capacity (DPPH) 175.78 ± 6.63 μmol TE/g dry matter. These results demonstrate that CVE‐AP enhanced anthocyanin recovery by 86% compared with atmospheric pressure extraction. UPLC‐UV profiling showed a flavonol‐rich extract dominated by rutin: 33.38 mg/g, achieving for ~67.7% of quantified phenolics. LC‐HRMS identified seven pigment‐related flavonoids, comprising anthocyanin and flavonol derivatives, including: Delphinidin‐3‐O‐(cis‐p‐coumaroyl‐glucoside), Kaempferol 3‐O‐(6ʹ‐O‐p‐coumaroyl)‐rutinoside, Cyanidin‐3‐O‐(p‐coumaroyl) glucose, Delphinin glucoside. CVE‐AP delivered significantly higher anthocyanin yields than atmospheric extraction, supporting a green and efficient route to valorize butterfly‐pea bioactives for functional food and nutraceutical applications.

## Graphical abstract

Closed‐vessel extraction under autogenous pressure (CVE‐AP) enhanced anthocyanin and phenolic compound recovery from *Clitoria ternatea* by improving solvent penetration and mass transfer while preserving flavonoid profiles confirmed by LC‐HRMS.

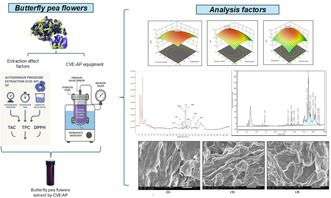

## 1 Introduction

*Clitoria ternatea* is an edible flowering plant widely used as a natural colorant in foods, pharmaceuticals, and bio‐based packaging (Vidana Gamage et al. 2021). *Clitoria ternatea* L. is widely cultivated throughout humid tropical regions worldwide, including India, Southeast Asia, Central and South America, the Caribbean, and Madagascar. Its precise geographical origin remains unclear, with differing accounts reported in the literature, and the historical native range of the species is considered difficult to determine due to extensive cultivation and naturalization (Mukherjee et al. 2008). Among its horticultural varieties, the blue‐flowered forms (*var. ternatea* and *var. pleniflora*) are of particular interest due to their intense and stable pigmentation (Havananda and Luengwilai 2019). Chemically, its phenolic profile is dominated by ternatin anthocyanins, and the flavonols kaempferol, quercetin, and myricetin (Jeyaraj et al. 2021; Vidana Gamage et al. 2021). The acylation and extensive glycosylation of these anthocyanins promote intramolecular copigmentation, which significantly enhances chromophore stability and underlies the characteristic deep blue hue of butterfly pea extracts. Phenolic compounds, including flavonoids and anthocyanins, are widely distributed plant secondary metabolites recognized for their antioxidant and protective biological activities (Luna‐Guevara et al. 2018). Chemically, phenolics contain at least one hydroxyl group directly attached to an aromatic ring, while polyphenols comprise multiple phenolic units and are generally classified into flavonoids and non‐flavonoids (Cosme et al. 2020). Among flavonoids, anthocyanins are water‐soluble glycosides localized in vacuoles, conferring red, purple, and blue pigmentation to plant tissues (Dangles et al. 1993). Their coloration highly depends on structural features and environmental factors, particularly pH, copigmentation, temperature, UV radiation, and oxygen, resulting in hues ranging from pink–red to deep purple–blue (Bridle and Timberlake 1997).

Mechanistically, anthocyanins mitigate reactive oxygen species (ROS) primarily through two pathways: hydrogen atom transfer (HAT) and single electron transfer (SET). In the HAT pathway, the antioxidant donates a complete hydrogen atom—a proton and an electron—to a radical, stabilizing it while generating a less reactive antioxidant‐derived radical, thereby slowing the overall oxidation process. In the SET pathway, the radical instead accepts a single electron from the antioxidant, becoming stabilized while converting the antioxidant into a transient radical cation intermediate (Chen et al. 2020).

Conventional extraction techniques for recovering anthocyanins and phenolics, such as maceration and Soxhlet extraction, often require long processing times and large volumes of organic solvents, which limits their sustainability and industrial applicability. To address these drawbacks, green extraction technologies have been increasingly explored. Pressurized liquid extraction (PLE), also known as accelerated or pressurized solvent extraction, has attracted attention due to its ability to enhance extraction efficiency through elevated temperature and pressure, improving solvent penetration and mass transfer while maintaining the solvent in a liquid state (Barp et al. 2023; Oro et al. 2025). Recent studies have demonstrated the effectiveness of PLE in recovering anthocyanins from various plant sources. For instance, PLE achieved extraction yields of approximately 80% (2.33 mg cyanidin‐3‐*O*‐glucoside equivalents (C3GE)/g) from black rice bran, outperforming conventional heating–stirring methods while preserving thermal stability and cytoprotective activity (Leonarski et al. 2023). In purple sweet potato, PLE enabled rapid and accurate quantification of anthocyanins, showing superior performance compared to traditional techniques (Truong et al. 2012). Similarly, optimized PLE conditions for açaí fruit facilitated efficient recovery of anthocyanins and total polyphenols, while minimizing solvent consumption (Aliaño‐González et al. 2020). Moreover, PLE has been employed to extract anthocyanin‐rich fractions from cracked black bean seed coats, yielding extracts with strong antioxidant activity using environmentally friendly solvents and reduced energy inputs (Teixeira et al. 2021).

Closed‐vessel extraction under autogenous pressure (CVE‐AP) has recently emerged as a promising alternative that addresses several limitations of conventional PLE. In CVE‐AP, internal pressure is generated passively by heating a sealed vessel, eliminating the need for externally applied pressure while still enhancing solvent diffusion and mass transfer (Cheng et al. 2021; Zhang et al. 2018). Building on this principle, the present study employed CVE‐AP to recover anthocyanins and polyphenols from *Clitoria ternatea* flowers, representing a novel application of this method for extracting bioactive compounds from butterfly pea. The research focused on optimizing the extraction conditions for anthocyanins and polyphenols using PLE, while simultaneously investigating the interactive effects of temperature and ethanol concentration as a passive pressure‐regulating mechanism within the closed system. The resulting extracts were evaluated for total anthocyanin content (TAC), total polyphenol content (TPC), and antioxidant capacity (DPPH). Furthermore, advanced analytical techniques, including scanning electron microscopy (SEM), high‐performance liquid chromatography (HPLC), and high‐resolution mass spectrometry (LC‐HRMS), were employed to elucidate cell wall disruption mechanisms and characterize the compositional profiles of the recovered compounds. Overall, the findings are expected to contribute to developing refined extraction protocols for bioactive compounds from natural resources, providing a scientific basis for their potential application in functional foods and pharmaceuticals.

## 2 Materials and Methods

### 2.1 Butterfly Pea Flowers

Fresh *Clitoria ternatea* flowers were collected between 06:00 and 08:00 AM from D'PALFARM Co. Ltd. (11°35′25″ N, 106°9′24″ E), located in Tay Ninh Province, Vietnam. The plant species was taxonomically identified at the Institute of Tropical Biology (Vietnam Academy of Science and Technology). After collection, the flowers were dried using a heat pump dryer at 40°C until the moisture content reached 12%–13% and stored in cool temperature for further extraction.

### 2.2 Method

#### 2.2.1 TPC, TAC, DPPH

Total phenolic content (TPC). TPC was quantified by the Folin–Ciocalteu colorimetric assay (ISO‐14502 2005). Appropriately diluted extracts were reacted with Folin–Ciocalteu reagent and Na_2_CO_3_ absorbance was read at 765 nm. Results are expressed as mg gallic acid equivalents (GAE) per g dry weight.

Total anthocyanin content (TAC). TAC was determined by the differential pH differential method (Giusti and Wrolstad 2001). Aliquots were diluted in pH 1.0 (KCl–HCl) and pH 4.5 (sodium acetate) buffers, and absorbance at 520/700 nm was recorded. Concentrations were calculated using the molecular weight and molar absorptivity of cyanidin‐3‐glucoside and reported as mg C3G equivalents g^−1^ dw.

DPPH radical‐scavenging activity. Antioxidant activity was assessed with the DPPH assay following (Brand‐Williams et al. 1995). Samples were incubated with a 0.1 mM methanolic DPPH solution; decolorization was measured at 517 nm after dark incubation. Data are expressed as μmol Trolox equivalents (TE) g^−1^ dw.

#### 2.2.2 Ultra‐Performance Liquid Chromatography (UPLC) Analysis

Anthocyanin profiling was performed using a Dionex Ultimate 3000 high‐performance liquid chromatography (HPLC) system (Thermo Finnigan, San Jose, CA, USA) coupled with a Q Exactive Hybrid Quadrupole‐Orbitrap Mass Spectrometer (Thermo Finnigan, San Jose, CA, USA). Chromatographic separation was carried out on a Luna C18 column (150 mm × 2.00 mm, 5 μm particle size; Phenomenex, USA) maintained at 30°C. The mobile phase consisted of solvent A (acetonitrile) and solvent B (0.05% v/v formic acid in water), with a gradient elution program as follows: 0–10 min, 98% B; 10–18 min, 80% B; 18–25 min, 40% B; and 25–30 min, re‐equilibration to 98% B. The flow rate was set to 0.3 mL/min. All data acquisition and processing were conducted using Thermo Scientific Xcalibur software.

#### 2.2.3 Q‐Orbitrap Mass Spectrometry Conditions

The Q Exactive mass spectrometer was operated in positive electrospray ionization (ESI) mode with a full MS scan range of *m/z* 100–1500 and a resolution of 70,000 (FWHM). Nitrogen was used as the sheath and auxiliary gas. The HESI parameters were as follows: sheath gas flow rate, 30 arbitrary units (arb); auxiliary gas, 10 arb; sweep gas, 0 arb; S‐lens RF level, 50 V; capillary temperature, 320°C; and auxiliary gas heater temperature, 300°C. MS/MS fragmentation was performed using higher‐energy collisional dissociation (HCD) with a normalized collision energy (NCE) of 20. Nitrogen was also used as the collision and spray stabilization gas within the C‐trap. Identification of anthocyanin compounds was based on their fragmentation patterns in comparison with previously reported reference spectra. Semi‐quantitative analysis was performed by calculating the relative abundance of individual anthocyanins as the percentage of their respective peak areas in the total ion chromatogram (TIC).

### 2.3 Chromatographic and Mass Spectrometric Analysis

#### 2.3.1 UPLC Conditions

Chromatographic separation of anthocyanins and phenolic compounds was performed using a Dionex Ultimate 3000 ultra‐performance liquid chromatography (UPLC) system (Thermo Fisher Scientific, San Jose, CA, USA). Samples were injected onto a Luna C18 column (150 mm × 2.00 mm, 5 μm; Phenomenex, Torrance, CA, USA), which was maintained at 30°C. The mobile phase consisted of solvent A (acetonitrile) and solvent B (0.05% v/v formic acid in water), delivered at a flow rate of 0.30 mL min^−1^ using a gradient elution program optimized for anthocyanin separation.

#### 2.3.2 LC‐HRMS Conditions

The UPLC system was coupled to a Q Exactive Hybrid Quadrupole‐Orbitrap mass spectrometer (Thermo Fisher Scientific, San Jose, CA, USA) equipped with a heated electrospray ionization (HESI) source and operated in positive ion mode. Full‐scan mass spectra were acquired over an m/z range of 100–1500 at a resolving power of 70,000 (FWHM at m/z 200). Nitrogen was used as sheath gas (30 arbitrary units), auxiliary gas (10 arbitrary units), and sweep gas (0 arbitrary units). The HESI source parameters were set as follows: capillary temperature 320°C, auxiliary gas heater temperature 300°C, and S‐lens RF level 50 V.

Tandem mass spectrometry (MS/MS) experiments were conducted using higher‐energy collisional dissociation (HCD) with a normalized collision energy (NCE) of 20. Nitrogen was used as the collision gas in the C‐trap and for spray stabilization.

#### 2.3.3 Data Processing and Compound Assignment

Data acquisition and processing were carried out using Thermo Scientific Xcalibur software. Anthocyanin and phenolic compounds were tentatively identified based on accurate mass measurements, MS/MS fragmentation patterns, and comparison with literature data and previously reported reference spectra. Semi‐quantitative analysis was performed by expressing the relative abundance of individual anthocyanins as the percentage of their respective peak areas relative to the total ion chromatogram (TIC).

### 2.4 Vapor–Liquid Equilibrium Modeling and Experimental Validation of Headspace Pressure

Internal vessel pressure was quantified during ethanol–water extraction experiments to characterize solvent behavior under elevated temperatures and to establish process safety parameters. Headspace pressure was modeled and validated using a sealed 500 mL stainless steel bottle containing 300 mL extract and a 200 mL air headspace initially at 25°C and 1 atm. Pressure predictions were obtained from Raoult's law. They extended Raoult's law with activity coefficients derived from published NRTL/UNIQUAC* parameters, while the contribution of entrapped air was estimated from the ideal gas law.

*Abbreviations*: NRTL, non‐random two‐liquid; UNIQUAC, universal quasi‐chemical. *Units*: Pressures are reported as absolute (psia) unless noted. Gauge readings (psig) were converted using psia = psig + 14.7 at 25°C.

#### 2.4.1 Pressure Modeling

Ethanol concentrations (% v/v) were converted to mole fractions using tabulated densities at 25°C (ρ_ethanol_ = 0.7893 g mL^−1^; ρ_water_ = 0.9970 g mL^−1^) and molar masses (*M*_ethanol_ = 46.068 g mol^−1^; *M*_water_ = 18.015 g mol^−1^). Pure‐component saturation pressures were estimated from the Antoine equation (temperature in °C), with parameters *A*_e_ = 8.20417, *B*_e_ = 1642.89, *C*_e_ = 230.3 for ethanol and *A*_w_ = 8.07131, *B*_w_ = 1730.63, *C*_w_ = 233.426 for water, which reproduce standard vapor pressure data between 1°C and 100°C (Shea 2000).

- *Model 1 (Ideal Raoult's law)*: Equilibrium vapor pressure was calculated as follows:

$$P_{v}=x_{\text{EtOH}}P_{\text{EtOH}}^{*}+\left(1-x_{\text{EtOH}}\right)P_{H_{2}O}^{*}$$

- *Model 2 (Extended Raoult's law)*: To capture positive deviations in ethanol–water mixtures, activity coefficients (*γ*) were incorporated:

$$P_{v}=x_{\text{EtOH}}\gamma_{\text{EtOH}}P_{\text{EtOH}}^{*}+\left(1-x_{\text{EtOH}}\right)\gamma_{H_{2}O}P_{H_{2}O}^{*}$$

with representative values of *γ*_EtOH_ = 1.25–1.35 and $\gamma_{H_{2}O}$ = 1.35–1.55 at 60°C–90°C, based on published NRTL/UNIQUAC parameters (Gmehling et al. 1982).

The contribution of entrapped headspace air was estimated using the ideal gas law:

$$P_{\mathrm{air}}\;\left(T\right)=P_{0}\times T/T_{0}$$

where *P*_0_ = 14.70 psi at *T*_0_ = 298.15 K. Total internal pressure was obtained by Dalton's law as:

$$P_{\text{total}}=P_{\mathrm{air}}+P_{v}$$

Experimental validation: Actual internal pressures were measured using a Bourdon‐type pressure gauge fitted to the extraction vessel, with calibration performed against compressed air standards (0–60 psig, referenced to atmospheric pressure). Measured values at 50°C–90°C were compared with model outputs, providing experimental validation of ethanol–water non‐ideal behavior and confirming the reliability of the predictive framework.

The pressure modeling was employed to estimate the range of autogenous pressures generated by different temperature–ethanol combinations in the closed‐vessel system. These estimates were used to define a safe and relevant operational window, guide experimental design, and enable indirect pressure control in the absence of externally applied pressure. As a result, all experiments were conducted below the pressure tolerance of the vessel while ensuring reproducible CVE‐AP conditions. Prior to experimentation, the pressure gauge, extraction vessel, and safety valve were formally verified and calibrated by an accredited metrology and inspection authority, confirming compliance with national technical and safety standards for pressure equipment. This verification ensured the accuracy of pressure measurements and the operational safety of the closed‐vessel system throughout the study.

The schematic representation below illustrates the pressure‐assisted extraction process of *Clitoria ternatea* flowers.

### 2.5 Extraction Process (CVE‐AP)

#### 2.5.1 Equipment and Operation

A closed‐vessel stainless‐steel chamber was employed for extraction, placed in a thermostatic water bath to maintain the desired temperature. Internal pressure was monitored simultaneously using a pressure sensor and a Bourdon gauge. After sample loading, the vessel was tightly sealed, and heating was initiated until the set temperature was reached. Extraction time was recorded once the bath temperature stabilized.

#### 2.5.2 Sample Preparation

Ten grams of dried *Clitoria ternatea* flowers were ground for 90 s and thoroughly mixed with a pre‐prepared ethanol–water solution at the designated ethanol concentration and liquid‐to‐solid (L/S) ratio. The solvent volume was calculated as:

$$V_{\text{solvent}}=\left(L/S,\mathrm{mL}\;g^{-1}\right)\times 10\;g$$

#### 2.5.3 Experimental Design

The three factors—autogenous pressure, extraction time, and liquid‐to‐solid ratio—were selected as key parameters directly influencing solvent penetration, mass transfer, and the recovery efficiency of anthocyanins and polyphenols. Baseline conditions were established based on preliminary experiments and literature data, under which one parameter was varied while the others were kept constant to evaluate its individual effect on extraction efficiency. A single‐factor experimental design was applied as follows:

- Autogenous pressure (AP): indirectly controlled by varying temperature (50°C–90°C) and ethanol concentration (55%–75% v/v), while extraction time and liquid‐to‐solid (L/S) ratio were fixed at 30 min and 18:1 mL/g, respectively.
- Extraction time (0–60 min): evaluated at fixed conditions of 80°C (corresponding to approximately 25–33 psi autogenous pressure), ethanol concentration of 60% v/v, and an L/S ratio of 18:1 mL/g.
- Liquid‐to‐solid ratio (14:1–22:1 mL/g): investigated at constant temperature (80°C), ethanol concentration (60% v/v), and extraction time (30 min), under conditions previously associated with high anthocyanin recovery.

Extraction under optimal conditions of temperature, time, ethanol content, solvent/raw material ratio, atmospheric pressure. Each condition was carried out in triplicate (*n* = 3). Parameters not under investigation were kept constant as specified above.

### 2.6 Statistical Analysis

All experiments were conducted in triplicate (*n* = 3). Results are expressed as mean ± standard deviation (SD). Prior to statistical analysis, data were inspected for normality and homogeneity of variances, and no outliers were excluded. Differences among treatments were evaluated using one‐way analysis of variance (ANOVA). When significant effects were detected (*p* < 0.05), mean comparisons were performed using a multiple range post hoc test. Statistical analyses were carried out using Statgraphics Centurion 19 (64‐bit version).

### 2.7 Chemicals and Equipments

#### 2.7.1 Chemical

Chemicals were purchased following: Gallic acid (Acros, Belgium), folin–ciocalteu (Sigma—Aldrich, USA), sodium carbonate (Xilong, China), DPPH (2,2‐diphenyl‐1‐picrylhydrazyl) (Acros, Belgium), Trolox (Sigma—Aldrich, USA), sodium acetate (Xilong, China), potassium chloride (Xilong, China), concentrated hydrochloric acid (Xilong, China), HCl (Sigma—Aldrich, USA), NaOH (Sigma—Aldrich, USA).

#### 2.7.2 Equipments

**FIGURE 1:**
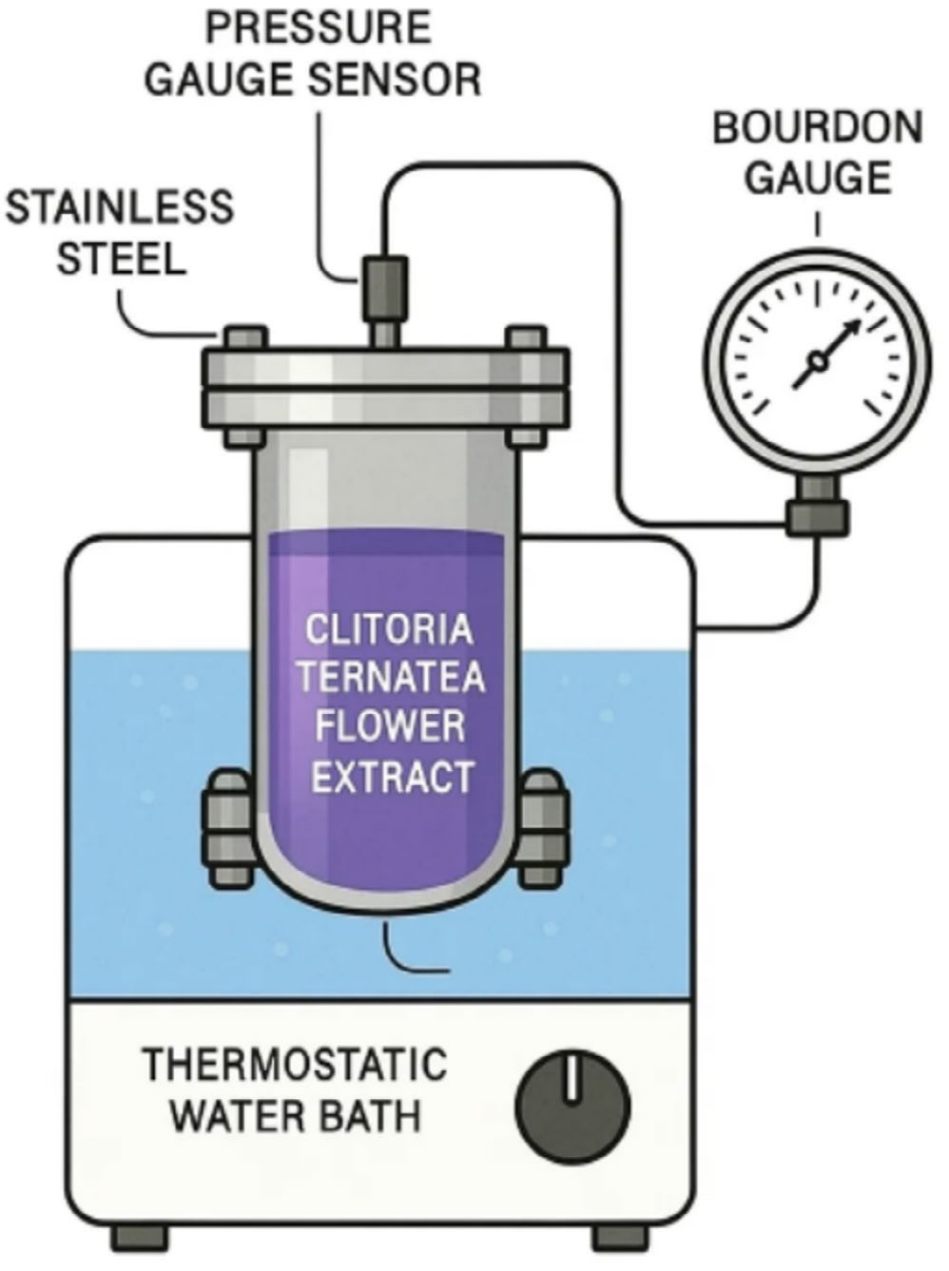
Schematic of the closed‐vessel, autogenous‐pressure (CVE‐AP) extraction setup used for *Clitoria ternatea* extraction, illustrating the sealed reactor, temperature control, and internal pressure monitoring.

Infrared Moisture Analyzer (ML‐50, AnD, Japan), convective drying (UM400, Memmert, Germany), pH machine (F20, Mettler Toledo, USA), thermostatic water bath (WNE‐29, Memmert, Germany), UV—Vis Spectrophotometer 25‐1650‐01‐0406, vacuum pump (RV8, Edwards, Germany), Labconco FreeZone Freeze Dry System (USA), Q Exactive Hybrid Quadrupole‐Orbitrap Mass Spectrometer (Thermo Finnigan, San Jose, CA, USA). The CVE‐AP setup was designed as illustrated in Figure 1.

## 3 Results and Discussion

### 3.1 Effects of Autogenous Pressure, Extraction Time, and Liquid‐to‐Solid Ratio on TAC, TPC, and Antioxidant Capacity (DPPH) of *Clitoria ternatea* Extracts

#### 3.1.1 Effect of Autogenous Pressure

Table 1 summarizes the estimated headspace pressures of ethanol–water mixtures, predicted by the Raoult–Antoine models (ideal and extended) as described in the Methods section. These predictions provide the theoretical range of autogenous pressures expected during extraction and serve as a benchmark for comparison with experimental measurements.

**TABLE 1:** Estimated autogenous pressures of ethanol–water systems based on Raoult–Antoine models and comparison with experimental values.

No	*T* (°C)	EtOH (% v/v)	*x*_EtOH_	$P_{\text{EtOH}}^{*}$ (mmHg)	$P_{H_{2}O}^{*}$ (mmHg)	*P*_air_ (psi)	Model 1 (ideal)		Model 2 (extended)		Experimental pressures
							*P*_*v*_ (psi)	*P*_total_ (psi)	*P*_*v*_ (psi)	*P*_total_ (psi)	
T1	50	0	0.0	220.3	92.3	15.9	1.8	17.7	1.8	17.7	17.9 ± 0.1
T2	60	20	0.072	350.7	149.0	16.4	3.2	19.6	4.2	20.6	21.1 ± 0.2
T3	70	40	0.171	541.2	233.2	16.9	5.5	22.4	7.8	24.7	25.6 ± 0.4
T4	80	60	0.317	812.2	354.5	17.4	9.7	27.1	14.0	31.4	32.9 ± 0.3
T5	90	60	0.317	1188.4	525.3	17.9	14.2	32.1	20.6	38.5	40.1 ± 0.2

*Note:* Atmospheric pressure (*P*_atm_) ~ 14.7 psi (the initial pressure in the vessel before heating).

Measured pressures were within the same range as those predicted by the models.

**FIGURE 2:**
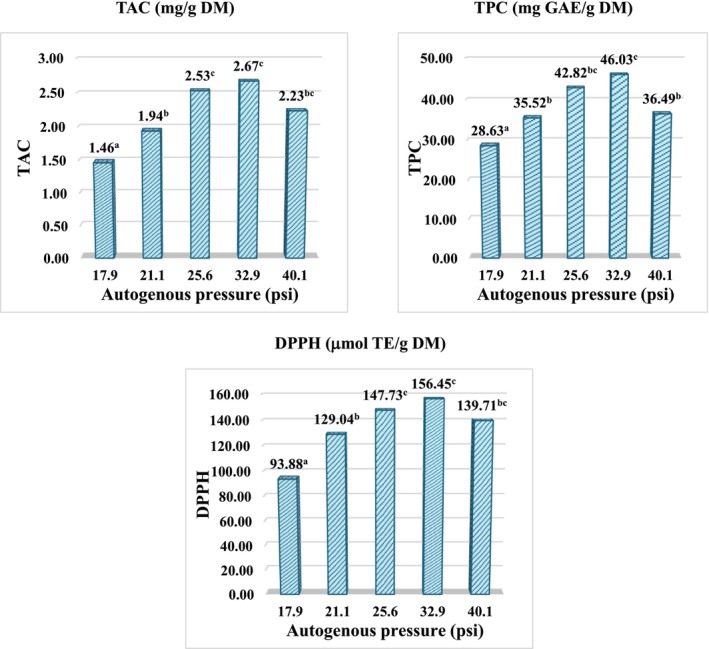
Effect of autogenous pressure on TAC, TPC, and antioxidant capacity (DPPH) of *Clitoria ternatea* flower extracts under closed‐vessel extraction conditions. Data is expressed as mean ± SD (*n* = 3). Different letters indicate significant differences among treatments determined by one‐way ANOVA followed by multiple range test (*p* < 0.05).

Figure 2 shows that, under closed‐vessel autogenous pressure (CVE‐AP) conditions, headspace pressure increased proportionally with temperature and ethanol–water composition, consistent with the principles described in Section 2.4 and the single‐factor design outlined in Section 2.5. In these experiments, extractions were performed at a fixed extraction time and liquid‐to‐solid ratio, while temperature and ethanol–water composition were systematically varied to generate different levels of autogenous pressure. The experimentally measured pressures, ranging from ~18 to 40 psi, fell within the predicted interval, thereby validating the reliability of the thermodynamic calculations for defining the operational domain (set points of temperature and ethanol mole fraction). From a performance perspective, TAC, TPC, and DPPH values increased markedly as autogenous pressure rose from ~18 to ~33 psi. This enhancement can be attributed to the combined effects of elevated temperature and pressure, which reduce solvent viscosity and surface tension, increase diffusivity, and promote solvent penetration into the plant matrix, thereby facilitating the release of anthocyanins and polyphenols. In ethanol–water systems, the adjustable polarity further improves solute–solvent affinity, enhancing extraction efficiency (Cheng et al. 2021; Huamán‐Castilla et al. 2023).

However, at ~40 psi, TAC, TPC, and DPPH values showed a slight decline, displaying an optimum‐then‐decrease response. At higher temperatures, the structural equilibrium of anthocyanins shifts from the colored flavylium cation toward hydrated hemiketal and chalcone forms (colorless), accompanied by increased thermal and oxidative degradation, thereby reducing the effective anthocyanin content and radical scavenging activity (Enaru et al. 2021; Reyes and Cisneros‐Zevallos 2007). Moreover, high ethanol fractions lower solvent polarity and water activity, decreasing the solubility of charged anthocyanins and potentially favoring the co‐extraction of components with limited antioxidant contribution (Huamán‐Castilla et al. 2023). This response pattern is consistent with previous reports on PHWE and PLE of anthocyanin‐rich matrices such as red onion, grape pomace, and grape skins, which similarly demonstrated maximal recovery at moderate temperature/pressure conditions followed by thermal fading at higher levels (Ju and Howard 2003; Peterson et al. 2010; Vergara‐Salinas et al. 2015).

This pressure‐dependent behavior provides a mechanistic basis for interpreting the effects of other operational parameters examined below.

#### 3.1.2 Effect of Extraction Time

**FIGURE 3:**
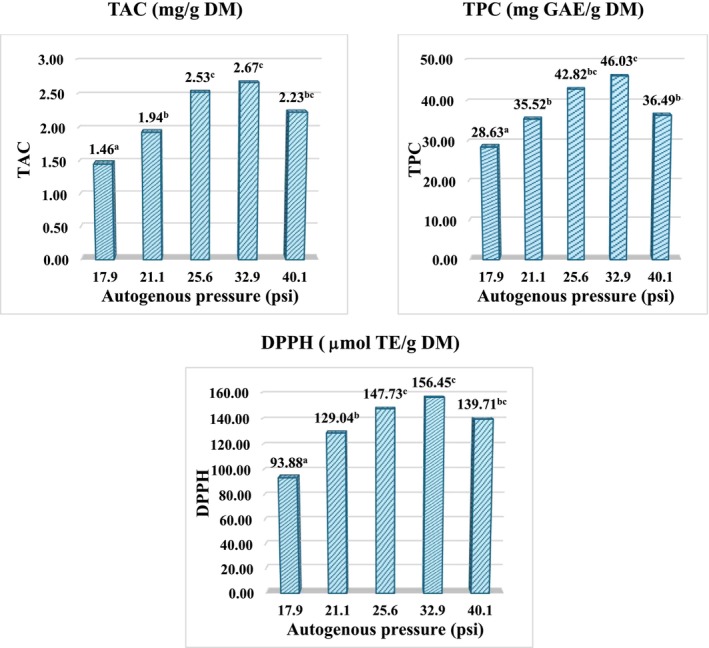
Effect of extraction time on TAC, TPC antioxidant capacity (DPPH) of *Clitoria ternatea* flower extracts. Data is expressed as mean ± SD (*n* = 3). Different letters indicate significant differences among treatments determined by one‐way ANOVA followed by multiple range test (*p* < 0.05).

The effect of extraction time on TAC, TPC, DPPH content is shown in Figure 3.

Figure 3 illustrates the influence of extraction time on TAC, TPC, and antioxidant capacity. All response variables increased rapidly during the initial 30 min, indicating that mass transfer and solvent penetration dominated the early extraction stage. Beyond this point, a gradual decline—particularly in TAC and DPPH—was observed at prolonged extraction times.

This time‐dependent response is consistent with the pressure‐driven optimum described in Section 3.1.1, where extended exposure to elevated temperature under closed‐vessel conditions promotes anthocyanin degradation and reduces effective antioxidant contribution. Similar optimum‐time profiles have been widely reported in pressurized extraction of anthocyanin‐rich plant matrices.

#### 3.1.3 Effect of Liquid‐to‐Solid Ratio

**FIGURE 4:**
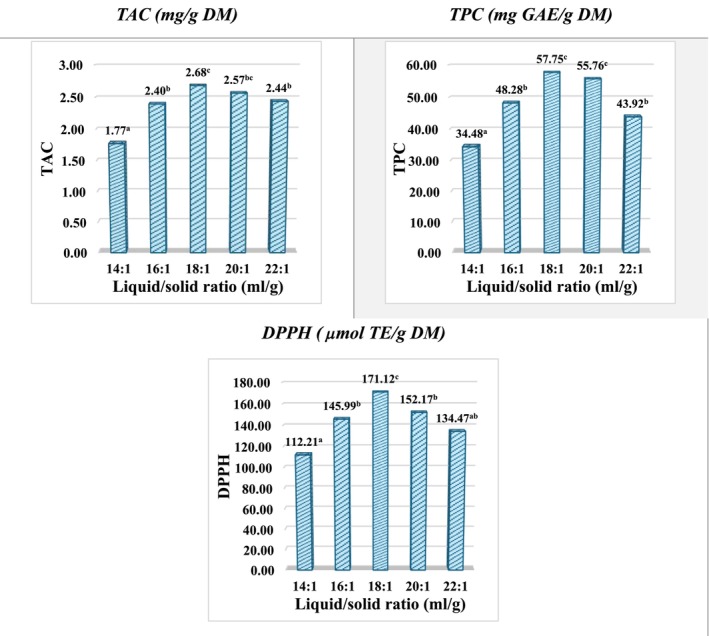
Effect of liquid‐to‐solid ratio on TAC, TPC, and antioxidant capacity (DPPH) of *Clitoria ternatea* flower extracts. Extractions were performed at fixed autogenous pressure and extraction time. Data are expressed as mean ± SD (*n* = 3). Different letters indicate significant differences among treatments determined by one‐way ANOVA followed by multiple range test (*p* < 0.05).

The influence of the liquid‐to‐solid ratio on TAC, TPC, and AC according to DPPH is presented in Figure 4.

As shown in Figure 4, increasing the liquid‐to‐solid ratio from 14:1 to 22:1 mL/g significantly enhanced TAC, TPC, and DPPH values, reflecting improved mass transfer driven by a higher concentration gradient between the solid matrix and the solvent. Further increases in solvent volume did not lead to additional improvements and resulted in a slight decline in all response variables. This suggests that once extraction equilibrium is reached, additional solvent does not shift the equilibrium but instead saturates the benefits of mass transfer and may even promote co‐extraction of compounds with low or negligible antioxidant activity or reduce solvent‐solute interaction efficiency (Cacace and Mazza 2006; Spigno et al. 2007).

This “increase‐to‐optimum followed by plateau or slight decrease” behavior mirrors the trends observed for autogenous pressure and extraction time (Section 3.1.1), suggesting that once extraction equilibrium is reached under CVE‐AP conditions, additional solvent primarily dilutes mass transfer efficiency and may favor the co‐extraction of compounds with limited antioxidant relevance. Comparable L/S‐dependent patterns have been consistently reported for polyphenol and anthocyanin extraction using green extraction technologies (Dai and Mumper 2010; Osorio‐Tobón 2020; Huamán‐Castilla et al. 2023).

Overall, the single‐factor experiments demonstrate that autogenous pressure acts as the dominant driving force governing extraction performance under CVE‐AP conditions, while extraction time and liquid‐to‐solid ratio modulate this effect within a narrower operational window. To further elucidate factor interactions and identify global optimum conditions, response surface methodology (RSM) was subsequently applied. To further contextualize the performance of CVE‐AP, its extraction efficiency and practical characteristics were qualitatively compared with other green extraction approaches previously reported for the same raw material. Previous studies on *Clitoria ternatea* have demonstrated that enzyme‐assisted extraction (EAE) and combined microwave–enzyme strategies can enhance the recovery of anthocyanins and phenolics. Specifically, EAE improved total phenolic yield by promoting cell wall disruption (Nguyen, Nguyen, et al. 2025), while the integration of microwave heating with enzyme assistance further accelerated extraction kinetics and increased antioxidant capacity through synergistic mechanical and biochemical effects (Nguyen, Le, Tran, et al. 2025).

Compared with these approaches, CVE‐AP achieves comparable or improved extraction performance without the need for externally applied pressure systems, enzymes, or microwave irradiation. By exploiting passively generated autogenous pressure to enhance solvent penetration and mass transfer, CVE‐AP offers advantages in terms of equipment simplicity, process reproducibility, and scalability. Nevertheless, alternative green extraction techniques remain relevant depending on specific process objectives and operational constraints.

### 3.2 RSM–CCD Optimization of TAC, TPC, and Antioxidant Capacity (DPPH)

Optimization was performed to identify suitable conditions for CVE‐AP of *Clitoria ternatea* . Single‐factor screening indicated that pressure, extraction time, and the liquid‐to‐solid ratio significantly affected the responses; these factors were therefore selected as independent variables for multivariate optimization. RSM was fitted using Stat‐Ease 360 with a three‐factor central composite design (CCD; 20 runs, including six center‐point replicates) executed in randomized order. Factors were coded (−α to +α) over ranges established in single‐factor tests. Model adequacy was evaluated by ANOVA, lack‐of‐fit testing, and *R* ^2^/*R* ^2^adj/*R* ^2^pred, with significance set at *p* < 0.05. The primary responses were TAC, TPC, and AC according to DPPH.

The experimental design matrix and the corresponding TAC responses obtained from the CCD experiments are presented in Table 2.

**TABLE 2:** Experimental results for total anthocyanin content.

Std. order	Run	X_1_ Autogenous pressure (psi)	X_2_ Time (min)	X_3_ Liquid/solid ratio (ml/g)	TAC (mg GAE/g DE)
16	1	32.9	30	16	2.71
13	2	32.9	30	14	2.49
5	3	25.6	15	18	2.02
10	4	40.2	30	16	2.15
6	5	40.2	15	18	1.79
20	6	32.9	30	16	2.72
3	7	25.6	45	14	2.55
8	8	40.2	45	18	1.62
19	9	32.9	30	16	2.72
2	10	40.2	15	14	2.11
4	11	40.2	45	14	1.92
11	12	32.9	15	16	2.19
15	13	32.9	30	16	2.69
18	14	32.9	30	16	2.68
14	15	32.9	30	18	2.27
1	16	25.6	15	14	1.98
9	17	25.6	30	16	2.59
12	18	32.9	45	16	2.38
17	19	32.9	30	16	2.69
7	20	25.6	45	18	2.32

ANOVA supported the quadratic RSM model for TAC (Table 3) with a highly significant model (*F* = 27.21, *p* < 0.0001; *R* ^2^ ~ 0.96). Among the main effects, autogenous pressure (A) contributed most (*p* < 0.0001), followed by the liquid‐to‐solid ratio (C) (*p* = 0.0062) and time (B) (*p* = 0.039). Of the two‐factor interactions, only A × B was significant (*p* = 0.001), whereas A × C and B × C were not (*p* > 0.05). All quadratic terms (A^2^, B^2^, C^2^) were significant (*p* < 0.05), consistent with the curvature observed in the response surfaces. The lack‐of‐fit test was significant (*p* = 0.0002), indicating that the quadratic form does not capture all systematic structure, despite the small pure error and good run‐to‐run reproducibility. Refinement could include selected cubic terms or a Box–Cox transformation to mitigate lack‐of‐fit.

**TABLE 3:** ANOVA for the quadratic RSM model of TAC.

Factor	SS	Df	MS	*F*	*p*
Model	2.17	9	0.2414	27.21	< 0.0001
A—Autogenous pressure (psi)	0.343	1	0.343	38.66	< 0.0001
B—Extraction time (minutes)	0.05	1	0.05	5.64	0.039
C—Liquid‐to‐solid ratio (ml/g)	0.1056	1	0.1056	11.9	0.0062
AB	0.1891	1	0.1891	21.32	0.001
AC	0.0231	1	0.0231	2.61	0.1376
BC	0.0078	1	0.0078	0.8806	0.3701
A^2^	0.0927	1	0.0927	10.45	0.009
B^2^	0.1985	1	0.1985	22.37	0.0008
C^2^	0.0829	1	0.0829	9.35	0.0121
Residual	0.0887	10	0.0089		
Lack of fit	0.0872	5	0.0174	58.81	0.0002
Pure error	0.0015	5	0.0003		
Cor total	2.26	19			

The regression equation for predicting TAC (mg/g dry matter) was:

(1) $$\begin{array}{cc} Y_{1}=\; & 2.64–0.19\times A+0.07\times B–0.10\times C–0.15\\ & \times \mathrm{AB}–0.05\times \mathrm{AC}–0.03\times \mathrm{BC}–0.18\\ & \times A^{2}–0.27\times B^{2}–0.17\times C^{2} \end{array}$$

*Y*_1_ is anthocyanin content – TAC (mg/g DM), A is autogenous pressure (psi), B is time (minutes), and C is LQ/SL ratio (ml/g).

Although the quadratic response surface model exhibited a high coefficient of determination, the significant lack‐of‐fit indicates that not all nonlinear behaviors of the extraction system were fully captured. Nevertheless, the pure error was low, and the model showed good predictive performance within the experimental domain, particularly around the optimal region. Furthermore, validation experiments confirmed close agreement between predicted and experimental responses, supporting the reliability of the identified optimal conditions for practical process optimization.

**FIGURE 5:**
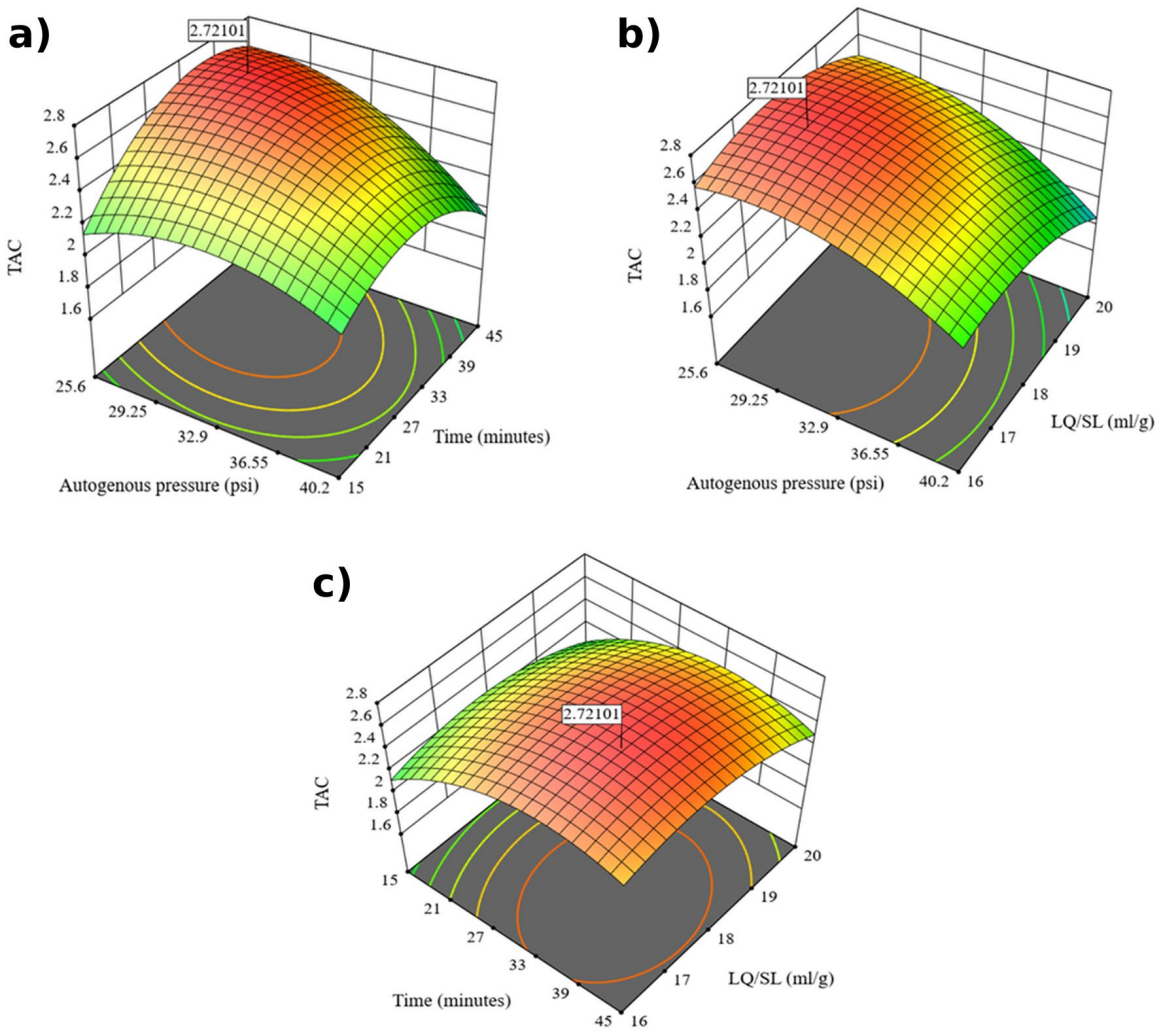
Three‐dimensional response surface and corresponding contour plot illustrating the correlation between factor pairs influencing TAC of *Clitoria ternatea* flower extracts.

Figure 5 shows that the response‐surface plots reveal nonlinear effects of autogenous pressure (A), extraction time (B), and the liquid‐to‐solid ratio (C) on TAC. The A–B surface shows a clear dome: TAC increases with both factors to intermediate levels and then declines, consistent with the significant A × B interaction in the ANOVA. In contrast, the A–C and B–C surfaces display curved profiles with maxima at mid‐range settings; these trends are driven primarily by quadratic terms (A^2^, B^2^, C^2^), while A × C and B × C interactions are not significant. Overall, curvature dominates the response, with A × B as the only meaningful two‐factor interaction. The highest predicted TAC (2.72 mg/g DM) occurs at intermediate levels of all three variables, indicating that extreme low or high settings are sub‐optimal for anthocyanin recovery.

Model‐based optimization identified 28.2 psi (A), 36 min (B), and 17.6 mL/g (C) (~18 mL/g (C)) as the optimum. Validation runs yielded TAC = 2.72 ± 0.18 mg/g DM, which did not differ from the prediction (*p* > 0.05). Under the same conditions, TPC = 56.82 ± 2.16 mg GAE/g DM, and DPPH = 175.78 ± 6.63 μmol TE/g DM. Compared with the atmospheric‐pressure control (TAC = 1.46 mg/g DM), the optimized CVE‐AP increased anthocyanin recovery by 86% (2.72 versus 1.46 mg/g DM). Therefore, despite the significant lack of fit, the model was considered sufficiently robust for practical optimization within the defined experimental domain.

### 3.3 Analytical Technique

#### 3.3.1 SEM

**FIGURE 6:**
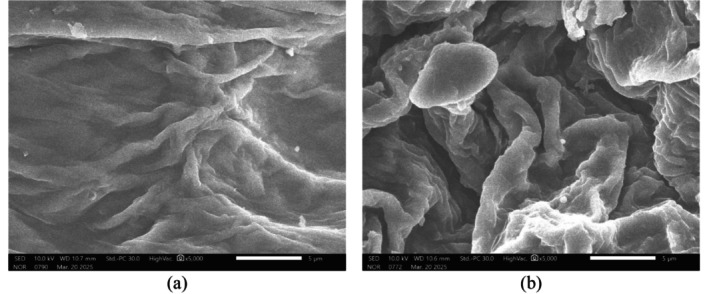
Scanning electron microscopy (SEM) images of *Clitoria ternatea* flower tissues illustrating cell wall morphology under different extraction conditions. (a) Atmospheric pressure extraction; (b) closed‐vessel extraction under autogenous pressure (CVE‐AP).

Figure 6 shows distinct microstructural differences between atmospheric pressure condition (a) and the sample treated under CVE‐AP (b). In the atmospheric‐pressure sample (a), the epidermal surface of the petals remained relatively intact, with cell layers arranged in parallel and appearing smooth; grinding for 90 s produced only shallow grooves without disrupting the cellulose–hemicellulose microfibrillar network, leaving the cell walls largely continuous. By contrast, after applying autogenous pressure, the cell walls appeared delaminated and fractured into fragments, with visible pores, microfissures, and stretched or curled cellulose microfibrils, resulting in a markedly more porous microstructure. Such damage is consistent with the “explosion–decompression” mechanism, in which hot solvent penetrates plant tissues under pressure, causing volumetric expansion; upon sudden pressure release, the cell‐wall matrix collapses. The more porous morphology and increased effective surface area are expected to shorten diffusion pathways and thereby enhance the release of anthocyanins and polyphenols into the solvent—consistent with previous reports on grape pomace subjected to high‐pressure treatment (Huamán‐Castilla et al. 2023).

Overall, the SEM observations demonstrate that CVE‐AP treatment induces more pronounced cell wall disruption and increased surface porosity compared to atmospheric pressure conditions, thereby facilitating solvent penetration and enhancing mass transfer. These microstructural alterations are consistent with the improved extraction efficiency observed and are further reflected in the compositional profiles of the extracts, as confirmed by HPLC and LC‐HRMS analyses.

#### 3.3.2 UPLC‐UV

**FIGURE 7:**
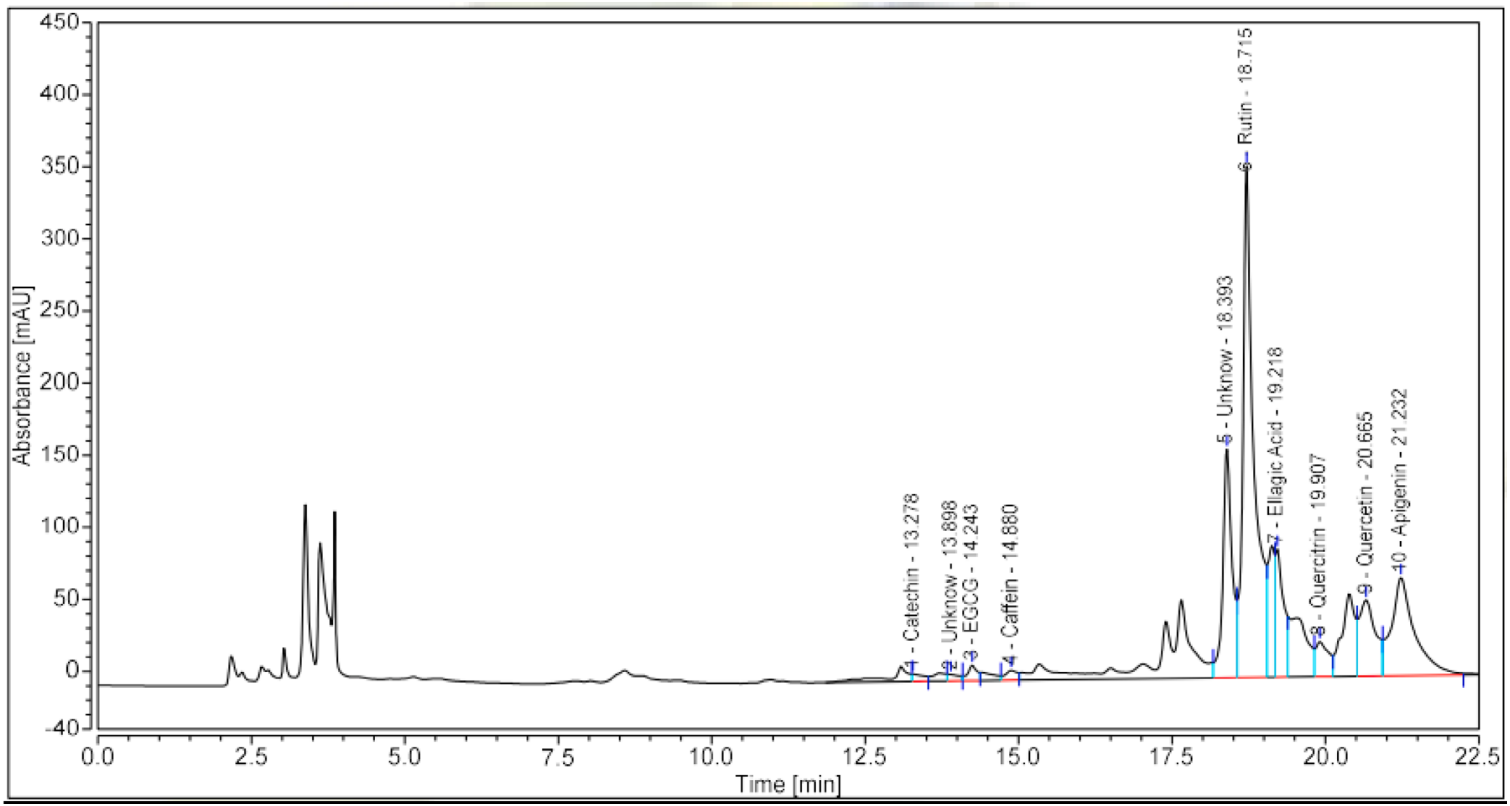
UPLC‐UV analysis of *Clitoria ternatea* extract components.

Table 4 shows that UPLC‐UV profiling identified eight major compounds in the *Clitoria ternatea* extract with a total quantified content of approximately 49.35 mg/g crude extract. Rutin was predominant (33.38 mg/g), accounting for ~67.7% of the total quantified amount, followed by apigenin (4.57 mg/g), quercetin (4.28 mg/g), catechin (2.39 mg/g), quercitrin (1.58 mg/g), ellagic acid (1.54 mg/g), EGCG (1.37 mg/g), and caffeine (0.249 mg/g). Chlorogenic acid was detected only at trace levels (~0.002 mg/g), while gallic acid was not detected within the LOD/LOQ limits. On the chromatogram, the target peaks were reasonably well resolved at their characteristic retention times (e.g., catechin ~13.28 min, EGCG ~14.26 min, caffeine ~14.88 min, rutin ~18.72 min, ellagic acid ~19.22 min, quercetin ~20.67 min, apigenin ~21.23 min), consistent with identification by authentic standards. The high proportion of flavonols, particularly rutin, is consistent with the effects of PLE: elevated temperature and pressure reduce solvent viscosity and enhance diffusivity, thereby improving the recovery of less polar compounds. Mild hydrolysis under PLE conditions may also contribute to the release of aglycones such as quercetin and apigenin (Xi and Luo 2015). These quantitative results are supported by the UPLC–UV chromatogram shown in Figure 7, which illustrates the separation profile and relative abundance of the major phenolic constituents identified in the *Clitoria ternatea* extract.

**TABLE 4:** Composition of phenolic compounds in *Clitoria ternatea* extracts.

No.	Contents	Content (mg/g)
1	Gallic acid	Not detected
2	Catechin	2.39 ± 0.01
3	Chlorogenic acid	0.00 ± 0.00 (trace)
4	EGCG	1.37 ± 0.00
5	Rutin	33.38 ± 0.02
6	Ellagic acid	1.54 ± 0.01
7	Quercitrin	1.58 ± 0.01
8	Quercetin	4.28 ± 0.01
9	Apigenin	4.57 ± 0.01
10	Caffein	0.25 ± 0.00

Abbreviation: EGCG, epigallocatechin gallate.

This hypothesis will be further examined through LC–MS/MS by monitoring glycoside–aglycone pairs. In addition, several unidentified peaks suggest the presence of other polyphenolic constituents, so a full‐range LC‐HRMS/UV–Vis analyses will be valuable for completing the identification and quantification of these compounds.

#### 3.3.3 LC‐HRMS

**FIGURE 8:**
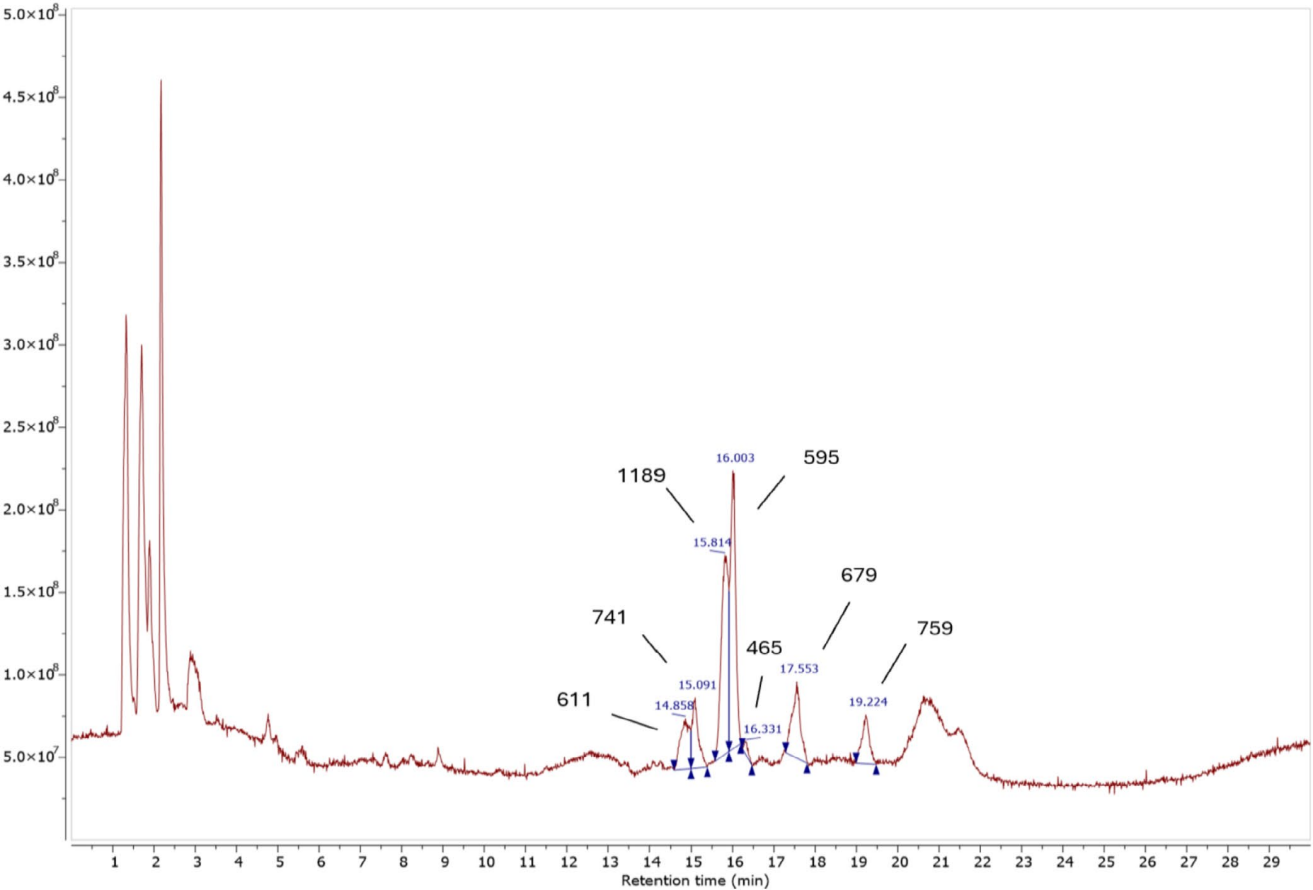
LC‐HRMS of butterfly pea flower extracted by autogenous pressure.

The LC‐HRMS analysis was conducted comparing the effect of different treatments on compositions of *Clitoria ternatea* . The identity of phytochemicals was verified based on their MS/MS fragmentation and comparison with literature data. The concentrations of *Clitoria ternatea* flower extracts were determined thanks to the percentage of peak area. The result is presented in Table 5, Figure 8, and supplementary of LC‐HRMS of butterfly pea flower extract by CVE‐AP. Seven compounds belonging to the anthocyanin group as well as aglycone of quercetin and kaempferol were identified in these treatments.

**TABLE 5:** Tentative anthocyanin composition of *Clitoria ternatea* extracts obtained by CVE‐AP based on LC‐HRMS analysis.

S#	Precursor (m/z, ESI+)	Tentative identification	Retention time (minutes)	Concentration (%)	MS^2^ fragment ions (m/z)	References
**S1**	**611** [M + H]^+^	Delphinidin‐3‐O‐(cis‐p‐coumaroyl‐glucoside)	14.858	8.80	465 [M + H‐146]^+^ 303 [M + H‐146–162]^+^	Bationo et al. (2023); Thuy, Ben, et al. (2021)
**S2**	**741** [M + H]^+^	Kaempferol 3‐O‐(6″‐O‐p‐coumaroyl)‐rutinoside	15.091	8.48	449 [M + H‐146‐146]^+^ 287 [M + H‐146‐146‐162]^+^	Kazuma et al. (2003); Tatsuzawa et al. (2014)
**S3**	**1189** [M + H]^+^	Unknown (could correspond to kaempferol derivative)	15.814	30.24	449, 287, 271, 182	
**S4**	**595** [M + H]^+^	Cyanidin‐3‐O‐(p‐coumaroyl)glucose	16.003	32.20	449 [M + H‐146]^+^ 287 [M + H‐146–162]^+^	Thuy, Minh, et al. (2021)
**S5**	**465** [M + H]^+^	Delphinin glucoside	16.331	1.04	303 [M + H‐162]^+^	Kazuma et al. (2003)
**S6**	**679** [M + H]^+^	Unknown (could correspond to cyanidin aglycone)	17.553	12.00	679, 404, 287, 212, 147	
**S7**	**759** [M + H]^+^	Unknown (no previous data has been reported)	19.224	7.24	741, 359, 331, 212	

Compound 1 presented [M + H]^+^ at m/z 611 and yielded similar ion fragmentation to compound 1 of m/z at 303. However, the mass spectrum showed the presence of a fragment ion m/z 147, corresponding to coumaroyl. Thus, compared with the data and the findings of (Thuy, Minh, et al. 2021), compound 1 was suggested as Delphinidin‐3‐O‐(p‐coumaroyl‐glucoside) (Bationo et al. 2023; Thuy, Minh, et al. 2021). Compound 2 was tentatively annotated Kaempferol 3‐O‐(6″‐O‐p‐coumaroyl)rutinoside due to showing protonated ion m/z at 741 and producing some fragments such as m/z 449, 287, evidencing the loss of two of neutral ions (146 Da) and glucoside group (162 Da). In addition, fragment ion 147 Da suggested this compound contained coumaroyl (Kazuma et al. 2003; Tatsuzawa et al. 2014). Compound 3 exhibited a protonated molecular ion 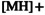 at m/z 1189 and produced several characteristic fragment ions at m/z 449, 287, 271, and 182. The fragment at m/z 287 could correspond to the aglycone cyanidin or kaempferol, which is consistent with a neutral loss of 162 Da from the fragment at m/z 449, similar to the fragmentation behavior observed for compound 4. However, the available data were insufficient to unambiguously determine the identity of the compound with a molecular weight of 1189. Compound 4 showed protonated ion with [M + H] + m/z 595, which was suggested as Cyanidin‐3‐O‐(p‐coumaroyl)glucose. Moreover, its fragments m/z 449, 287, 147 revealed this compound involving coumaroyl (147 Da), glucoside (loss of neutral ion [M + H146‐162]^+^), and aglycone cyanidin (287 Da) (Thuy, Minh, et al. 2021).

Compound 5 showed [M + H]^+^ m/z at 465 and produced main ion at m/z 303, resulting in the loss of 162 Da. This compound was tentatively identified as Dephinin glucoside (Kazuma et al. 2003). Compound 6 exhibited a protonated molecular ion [M + H]^+^ at m/z 679 and generated fragment ions at m/z 404, 287, 212, and 147. The fragment at m/z 287 may correspond to the aglycone cyanidin or kaempferol. Compound 7 presented a [M + H]^+^ ion at m/z 759 and produced fragment ions at m/z 741, 359, 331, and 212; however, no previous data have been reported to support the identification of this compound. To date, no relevant literature evidence has been found to substantiate the structural assignment of these two compounds. Consequently, their identities remain inconclusive, and further analytical studies are required to precisely elucidate their chemical structures.

All compounds were tentatively identified based on accurate mass measurements, MS/MS fragmentation behavior, and comparison with literature data. However, the absence of authentic reference standards precludes unambiguous structural confirmation. Supporting LC‐HRMS spectra and additional fragmentation data for the annotated compounds are provided in Figures S1–S7.

## 4 Conclusion

CVE‐AP proved to be highly effective in enhancing the recovery of anthocyanins and polyphenols from *Clitoria ternatea* flowers. Single‐factor analysis combined with response surface methodology (RSM–CCD) demonstrated that autogenous pressure, extraction time, and liquid‐to‐solid ratio were critical parameters significantly affecting TAC, TPC, and antioxidant capacity (DPPH) (*p* < 0.05). The optimal conditions were identified as an autogenous pressure of 28.2 psi, an extraction time of 36 min, and a solvent‐to‐solid ratio of 18:1 mL/g, yielding validated values of TAC 2.72 ± 0.18 mg/g DM, TPC 56.82 ± 2.16 mg GAE/g DM, and DPPH 175.78 ± 6.63 μmol TE/g DM. SEM observations confirmed cell‐wall disruption and increased micro‐porosity, while HPLC and LC‐HRMS analyses provided clear evidence of preserved and well‐defined phenolic and anthocyanin profiles, consistent with pressure‐enhanced mass transfer mechanisms. Compared with atmospheric pressure extraction, anthocyanin content increased by ~86%, underscoring the pivotal role of autogenous pressure in improving extraction efficiency. These findings highlight CVE‐AP as a promising “green” extraction technology for valorizing natural plant materials and open new perspectives for further research on extended pressure ranges, compound stability, process scalability, and applications in functional foods and pharmaceuticals. From a practical perspective, the CVE‐AP approach offers favorable scalability and industrial applicability, as it relies on simple closed‐vessel systems without the need for externally applied pressure, specialized enzymes, or microwave equipment. The moderate operating pressure and solvent conditions used in this study are compatible with conventional food‐grade processing infrastructure, facilitating scale‐up and process integration. These features make CVE‐AP particularly attractive to produce anthocyanin‐ and polyphenol‐rich extracts intended for functional food, nutraceutical, and natural colorant applications. Future work should further evaluate pilot‐scale performance, long‐term process stability, and economic feasibility under industrial conditions.

## Author Contributions

**Thi Thu Tra Tran:** investigation, methodology, validation, visualization, formal analysis, data curation. **Thi Anh Dao Dong:** conceptualization, methodology, investigation, validation, writing – review and editing, supervision, formal analysis. **Kha Duyen Nguyen:** investigation, writing – original draft, methodology, validation, software, formal analysis, data curation, resources.

## Funding

The authors have nothing to report.

## Conflicts of Interest

The authors declare no conflicts of interest.

## Supporting information


**Figures S1‐S7:** fsn371647‐sup‐0002‐FigureS1‐S7.docx.

## Data Availability

Supporting Information associated with this article are available in the online version. The Supporting Information file contains additional figures (Figures S1–S7) providing supporting data for the extraction experiments and LC–HRMS characterization of anthocyanin compounds from Clitoria ternatea extract.
