# Supplementary material for: Improvement of Phenolic Compound and Anthocyanin Extraction Efficiency From Clitoria ternatea by Autogenous Pressurization Method in a Sealed‐Vessel and Determination of Anthocyanin Compounds by LC‐HRMS
Source: Food Sci Nutr. 2026 Mar 15;14(3):e71649. doi: 10.1002/fsn3.71649 (PMC13093360; doi:10.1002/fsn3.71649)
Supplement: Supplementary file 1 — Figures S1‐S7: fsn371647‐sup‐0002‐FigureS1‐S7.docx. [file FSN3-14-e71649-s001.docx]

### Supplementary Materials (Figures S1–S7)

Supplementary Figures S1–S7 present the extracted ion chromatograms (EICs) and corresponding MS/MS fragmentation spectra used for the tentative identification of anthocyanin-related compounds in CTE by LC-HRMS.

Fragment ion chromatograms of precursor ions at m/z 611, 741, 1189, 595, 465, 679, and 759 are shown in Figures S1–S7, respectively. Identifications are tentative and based on high-resolution MS/MS data and comparison with literature reports.

All spectra were acquired in positive electrospray ionization (ESI+) mode, with protonated molecular ions reported as [M+H]⁺. Retention times (RT) are expressed in minutes.

| **Precursor (m/z, ESI+)** | **MS^2^ fragment ions (m/z)** |
| --- | --- |
| **611** |  |
|  | **Figure S1**:   - EIC (ESI+) at m/z 611 ([M+H]^+^), RT 14.235 min. - MS/MS: m/z 465, 303. - Tentative: Delphinidin-3-O-(cis-p-coumaroyl)glucoside. |
| **741** | 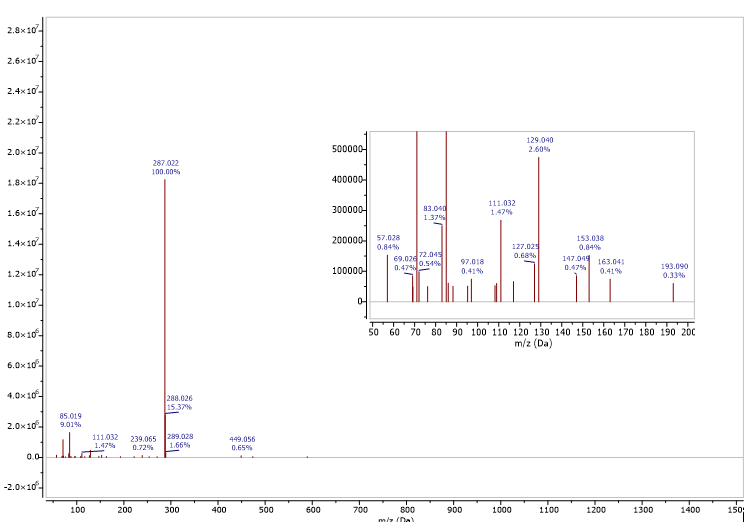 |
|  | **Figure S2:**   - EIC (ESI+) at m/z 741 ([M+H]^+^), RT 15.031 min. - MS/MS: m/z 449, 287. - Tentative: Kaempferol 3-O-(6’’-O-p-coumaroyl)rutinoside. |
| **1189** |  |
|  | **Figure S3***:*   - EIC (ESI+) at m/z 1189 ([M+H]^+^), RT 15.825 min. - MS/MS: m/z 449, 287, 271, and 182. - Tentative: Unknown (could correspond to kaempferol derivative) |
| **595** | 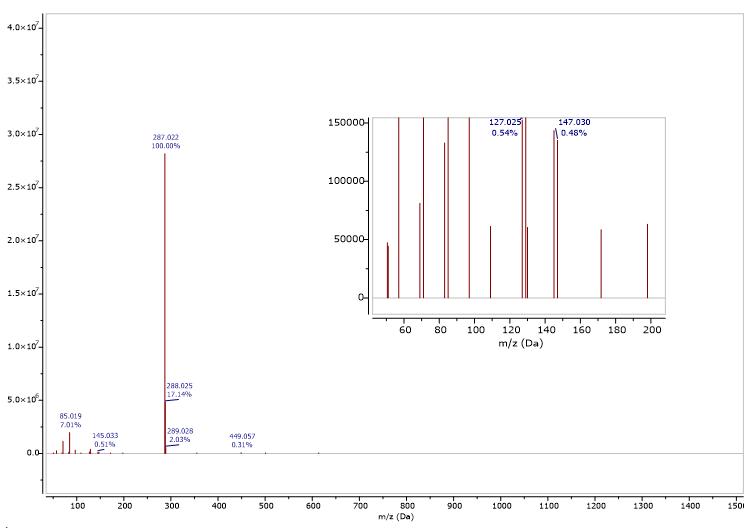 |
|  | **Figure S4**:   - EIC (ESI+) at m/z 595 ([M+H]^+^), RT 16.022 min. - MS/MS: m/z 449, 287. - Tentative: Cyanidin-3-O-(p-coumaroyl)glucose. |
| **465** |  |
|  | **Figure S5**:   - EIC (ESI+) at m/z 465 ([M+H]^+^), RT 16.331 min. - MS/MS: m/z 303, 287. - Tentative: Delphinin glucoside. |
| **679** |  |
|  | **Figure S6**:   - EIC (ESI+) at m/z 679 ([M+H]^+^), RT 17.485 min. - MS/MS: m/z 404, 287, 212, 147. - Tentative: Unknown (could correspond to cyanidin aglycone) |
| **759** |  |
|  | **Figure S7**:   - EIC (ESI+) at m/z 759 ([M+H]^+^), RT 18.72 min. - MS/MS: m/z 741, 359, 331, and 212. - Tentative: Unknown (no previous data has been reported). |
